# Rush Medical College Commencement

**Published:** 1874-03

**Authors:** 


Rush Medical College Commencement.—The thirty-third annual Commencement of the Rush Medical College was held on the evening of February 17, at the Michigan Avenue Baptist Church. The church was well filled with friends of the institution. The President, Dr. Freer, gave a brief history of the College since its organization, previous to the presentation of the diplomas to the graduating class. Degrees were conferred upon seventy-six graduates. Dr. Bennett delivered the valedictory in behalf of the class. Professor Miller then delivered the closing address. Dr. Mitchell, in behalf of his fellow-graduates, presented to Prof. Powell a handsome watch and chain. The exercises were pleasantly interspered with music by a band.

